# In Chronic Hepatitis C Infection, Myeloid-Derived Suppressor Cell Accumulation and T Cell Dysfunctions Revert Partially and Late After Successful Direct-Acting Antiviral Treatment

**DOI:** 10.3389/fcimb.2019.00190

**Published:** 2019-06-14

**Authors:** Valentina Telatin, Francesco Nicoli, Chiara Frasson, Nicola Menegotto, Francesco Barbaro, Eleonora Castelli, Elke Erne, Giorgio Palù, Antonella Caputo

**Affiliations:** ^1^Department of Molecular Medicine, University of Padova, Padova, Italy; ^2^Istituto di Ricerca Pediatrica (IRP) Città della Speranza, Padova, Italy; ^3^Infectious and Tropical Diseases Unit, Azienda Ospedaliera di Padova, Padova, Italy

**Keywords:** HCV, DAA, M-MDCSs, Tregs, T lymphocytes

## Abstract

Chronic HCV infection is characterized by several immunological alterations, such as the accumulation of suppressor cells and of hyperactivated T lymphocytes. However, it is unclear whether direct-acting antiviral (DAA)-mediated HCV clearance restores immune dysfunctions. We performed a phenotypic characterization by flow cytometry of different immune cell subsets, including monocytic myeloid-derived suppressor cells (M-MDSCs) and T lymphocytes in 168 patients with persistent HCV infection not treated, under DAA therapies and sustained virological responders. Chronic HCV infection prompted the accumulation of M-MDSCs independently of patient and clinical characteristics, and altered their metabolic properties. HCV RNA was undetectable in the majority of patients just after few weeks of DAA therapy, whereas M-MDSC levels normalized only 6 months after therapy. In addition, HCV infection deeply perturbed the T cell compartment since a re-distribution of memory CD4^+^ and CD8^+^ T cells was observed at the expenses of naïve cells, and memory T lymphocytes displayed increased activation. Notably, these features were only partially restored by DAA therapies in the CD4, but not in the CD8, compartment as high immune activation levels persisted in the terminally differentiated memory CD8^+^ T cells even more than 1 year after sustained virological response. Together, these results suggest that successful DAA therapies do not lead to full immunological reconstitution as fast as viral clearance.

## Introduction

The World Health Organization (WHO) estimates that more than 70 million people are chronically infected with hepatitis C virus (HCV), causing about 400,000 deaths every year, and that ~3–4 million new infections occur each year worldwide (WHO, 2018). Chronic HCV infection represents a global health challenge because it leads to liver fibrosis, cirrhosis and hepatocellular carcinoma (HCC). At present, although numerous candidates have been pursued, there is no vaccine available to prevent HCV infection, transmission, and eradication. New generation of highly effective interferon-free, direct-acting antiviral (DAA) therapies have revolutionized the care of HCV-infected individuals due to their dramatically high cure rate (Hezode, 2018). However, the low access to this new generation of drugs in low income countries, the detection in few patients of occult HCV infection (Attar and Van Thiel, 2015; Elmasry et al., 2017) and the emergence of drug resistance and suboptimal activity toward some genotypes (Sun et al., 2018; Tavares et al., 2018) occasionally reported in DAA-treated subjects could threaten the achievement of HCV eradication in the absence of an effective vaccine.

Several alterations of both innate and adaptive immunity also occur in chronically HCV-infected patients (Fernandez-Ponce et al., 2017), including increased level of myeloid-derived suppressor cells (MDSCs), that may inhibit T cell responses favoring viral escape and disease progression (Ning et al., 2015). Immunosuppressive myeloid cells are most likely generated as normal physiological response to acute and excessive inflammatory conditions (Bronte, 2009; Bronte et al., 2016). In healthy individuals MDSCs are present in low numbers in the blood, whereas they rapidly expand during pathological conditions such as cancer, autoimmune or infectious diseases, trauma, sepsis and bone marrow transplantation (Bronte, 2009; Bronte et al., 2016). Among the different subsets of MDSCs, it has been reported that the monocytic ones (M-MDSCs) accumulate more in HCV infection (Ning et al., 2015). However, little is known about the role of DAA therapies on the restoration of MDSC numbers.

Within this frame, a hallmark feature of persistent HCV infection is chronic immune activation and dysfunction of several types of immune cells, including naïve and memory CD4^+^ and CD8^+^ T cells, which have been linked to perturbation of anti-viral and anti-tumoral immune responses (Urbani et al., 2006; Alanio et al., 2015). These immune alterations may increase susceptibility of chronically infected patients to heterologous infections and their severe consequences (Marrie et al., 2017) and to extra-hepatic tumors (Pol et al., 2018) as well as may decrease responses to vaccination (Buxton and Kim, 2008). At present, it is unclear whether effective inhibition of HCV replication by DAAs influences immune activation and restores the immune functions and immune surveillance capacity in HCV-cured patients (Tumino et al., 2017; Guarino et al., 2018; Singh et al., 2018).

To gain further insights into the activity of IFN-free treatments on the immune dysfunctions, the main objective of this study was to evaluate the capacity of DAAs of reestablishing those cellular response features known to be affected by HCV infection and/or to be crucial for the effectiveness of adaptive immunity. In particular, we investigated the presence and quality of suppressor cell populations such as M-MDSCs and Tregs and the proportion as well as the activation and exhaustion phenotype of different CD4^+^ and CD8^+^ T cell subpopulations.

The main results of the study confirm that HCV infection deeply alters, quantitatively and qualitatively, both the myeloid and lymphoid compartment, and indicate that DAA-based therapies lead partially and slowly to restoration of these immunological alterations.

## Materials and Methods

### Study Design

HCV-chronically infected patients were enrolled for a cross-sectional study (*n* = 168) and for a nested longitudinal study (*n* = 11) at the Infectious and Tropical Disease Unit of the Azienda Ospedaliera of Padua after signing an informed consent. Patients with HIV or HBV co-infections, malignancy different from liver cancer, autoimmune diseases and pregnant women were excluded. The following information were provided: values of aspartate aminotransferase (AST) and alanine aminotransferase (ALT), blood HCV RNA load, HCV genotype, age, and sex (Table S1).

Patients undergoing therapy and sustained virological responders were treated with different combinations of DAA regimens (Table S2). HCV-negative healthy donors (*n* = 47) were also enrolled as healthy controls. The study was approved by the Ethics Committee of the Azienda Ospedaliera of Padua (Prot. n. 3136/AO/14) and conducted according to the principles expressed in the Declaration of Helsinki.

### Sample Purification

Whole peripheral blood was collected in spray-coated K2EDTA tubes. Briefly, for total leucocytes purification, blood was diluted 1:3 with hemolysis's solution [NH_4_Cl (8,6g/l), KHCO_3_ (1g/l), EDTA tetrasodium (0.037 g/l)], incubated for 20 min, and centrifuged at 1,200 rpm for 7 min. To remove residual red blood cells, the cellular pellet was further diluted with 10 ml of hemolysis's solution and processed as above. The pellet was resuspended in 10 ml of 1X Dulbecco's phosphate buffer saline without calcium and magnesium (1X D-PBS) (Gibco) and alive cells counted by the Trypan blue dye exclusion method. An aliquot of fresh total leukocytes was immediately analyzed by FACS.

PBMCs and plasma were obtained only from a restricted number of donors due to small volume of blood available. PBMCs were purified by Ficoll (GE Healthcare) density gradient, as previously described (Sforza et al., 2014; Nicoli et al., 2018). Cells were stored in 90% fetal bovine serum (FBS) (Lonza) and 10% DMSO (Sigma-Aldrich) in liquid nitrogen. For plasma collection, a whole blood tube (6 ml) was centrifuged at 2500 rpm for 7 minutes at room temperature. After centrifugation, 1 ml of plasma was centrifuged at 3,000 rpm for 5 min to remove residual cells and stored at −80°C.

### Flow Cytometry Analyses

The frequency and phenotype of MDSCs were analyzed by flow cytometry after surface staining in the dark for 15 min at room temperature with monoclonal antibodies (mAbs). The monocyte fraction of MDSCs (M-MDSCs, CD33^+^CD11b^+^HLA-DR^−/low^CD15^−^CD14^+^) was identified using the following fluorochrome-conjugated anti-human mAbs: anti-CD14 PE-Cy7 (eBioscience), anti-HLA-DR APC (eBioscience), anti-CD15 eFluor® (eBioscience), anti-CD33 FITC (eBioscience), anti-CD11b PE (Beckman Coulter) and expressed as percentage of PBMCs.

The characterization of the T cell subsets was performed using anti-CD4 APC (eBioscience), anti-CD3 BV605 (eBioscience), anti-CD8 APC-Cy7 (BD-biosciences), anti-HLA-DR PE-Cy7 (BD-biosciences), anti-CD38 PE-CF594 (BD-biosciences), anti-PD1 PerCP-Cy5.5 (BioLegend), anti-CD27 Alexa Fluor 700 (BioLegend), and anti-CD45RA V450 (BD-biosciences) mAbs. For Treg staining, cells were incubated with Foxp3 fixation/permeabilization working solution and the anti-FoxP3 FITC mAb (eBioscience) according to manufacturer's instructions.

The production of reactive oxygen species (ROS) and the mitochondrial membrane potential (ΔΨM) in M-MDSCs were assessed using the CellROX® Green Reagent (Life Technologies) and tetramethylrhodamine (TMRM, Life Technologies), as previously described (Nicoli et al., 2018). After washing, cells were stained with the specific fluorochrome-conjugated mAbs for M-MDSC identification: anti-CD15 eFluor450 (eBioscience), anti-CD33 Alexa Fluor700 (eBioscience), anti-HLA-DR APC (eBioscience), anti-CD14 PE-Cy7 (eBioscience), and anti-CD11b Viogreen™ (Miltenyi Biotec).

Data were acquired using a BD LSR II flow cytometer (BD Biosciences) and analyzed with FlowJo software (Tree Star).

### T Cell Suppression Assay

As a robust definition for human MDSC subsets is still lacking (Mandruzzato et al., 2016), the gating strategy was confirmed by analyzing the function of M-MDSCs sorted from PBMCs of representative subjects with FACSAria II cell sorter (BD). Heterologous PBMCs, from healthy donors, were labeled with a solution containing 5 μM 5(6)-carboxy-fluorescein diacetate succinimidyl ester (CFSE, eBioscience) for 10 min at 37°C and washed twice before culturing. Labeled PBMCs were seeded in 384-well plates (Falcon) coated with 80 μl of anti-human CD3 mAb (eBioscience) at 1 μg/ml and cultured without and with sorted M-MDSCs at 2:1 ratio. T cell proliferation was induced by soluble anti-human CD28 mAb (Miltenyi Biotec) at 0.1 μg/ml for 4 days at 37°C. Detached cells were transferred into FACS tubes, washed, stained with anti-human CD8 APC-Cy7 and analyzed by flow cytometry.

### Analysis of Cytokine Plasmatic Levels

Millipore's MILLIPLEX® MAP Human High Sensitivity T Cell Magnetic Bead Panel (Millipore) was used according to the manufacturer's instructions, with the Bio-Plex 200 System (BioRad) and the Bio-Plex Pro Wash Station (BioRad) as previously described (Borgatti et al., 2010; Lampronti et al., 2017).

### Statistical Analysis

Mann-Whitney and Wilcoxon signed-rank tests were used to compare the difference between two independent or paired groups respectively. The one-way analysis of variance (ANOVA) was used to determine any statistically significant differences between the means of more than three independent (unrelated) groups, and Bonferroni correction was used. Correlations were analyzed by Spearman's rank test. *P* values < 0.05 were considered to be statistically significant.

## Results

### Study Subjects

The baseline characteristics of the enrolled participants are summarized in Table S1. HCV-chronically infected patients (*n* = 168) were enrolled for the cross-sectional study and grouped in: not-treated patients (NT, *n* = 75), patients during pharmacological treatment with DAA (T, *n* = 53) and sustained virological responders (SVR, *n* = 40). The SVR group considered in this analysis, if not otherwise specified, includes different time points after sustained virological response: 25 subjects since 12 weeks (SVR12), 5 since 24 weeks (SVR24), 8 since 48 weeks (SVR48), and 2 since 96 weeks (SVR96) after the end of therapy. The majority of patients was infected with HCV genotype 1. HCV viral loads reached very low levels (<10^3^) already during treatment (group T), and the same trend was observed for AST and ALT values.

### M-MDSC Frequency Is Increased During Chronic HCV-Infection and Not Influenced by Virological and Clinical Characteristics

**Figure 1:**
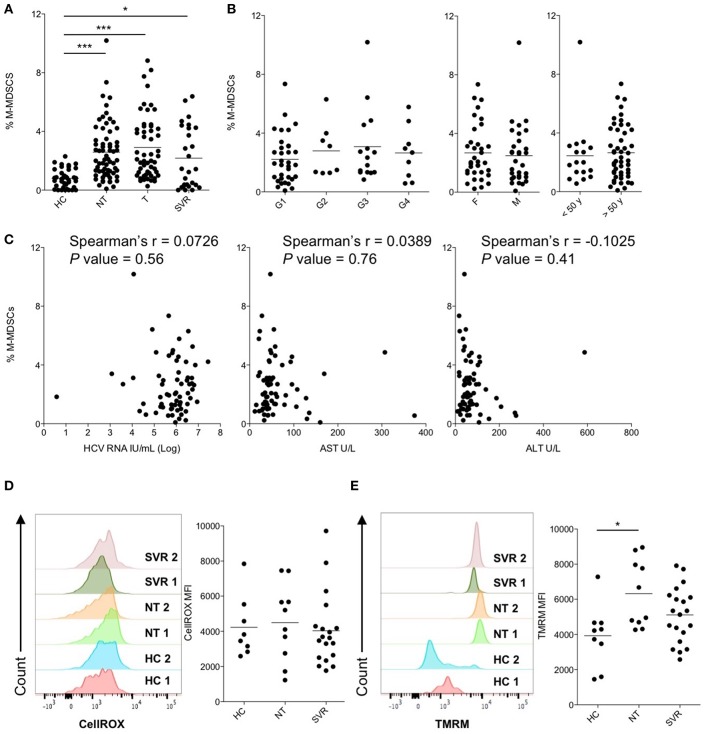
Cross-sectional analysis of M-MDSCs in peripheral blood of patients with untreated and treated HCV-chronic infection. **(A)** Percentage of M-MDSCs in healthy controls (HC, *n* = 38) and in subjects with HCV-chronic infection, not-treated (NT, *n* = 66), under therapy (T, *n* = 53), and sustained virological responders (SVR, *n* = 27). **(B)** Percentage of M-MDSCs in NT HCV-infected patients stratified by HCV genotypes (G1, *n* = 34; G2, *n* = 8; G3, *n* = 15; G4, *n* = 9), sex (*F* = female, *n* = 35; *M* = male, *n* = 31), and age (<50 y = below 50 years old, *n* = 17; >50 y = below 50 years old, *n* = 49). **(C)** Correlation between the percentage of M-MDSCs and peripheral blood HCV-RNA viral load, serum AST and ALT levels in NT HCV-infected patients (*n* = 66). **(D)** Content of ROS, expressed as CellROX MFI, in M-MDSCs of HC (*n* = 8) and of subjects with HCV-chronic infection, NT (*n* = 10), and SVR (*n* = 19). **(E)** ΔΨM expressed as TMRM MFI in M-MDSCs of HC (*n* = 9) and of subjects with HCV-chronic infection, NT (*n* = 10), and SVR (*n* = 20). **(A,B,D,E)** Lines represent the means. **(D,E)** Left panels show histograms from two representative donors for each group, whereas right panels show single donors. ^*^P < 0.05, ^***^P < 0.0001 calculated one-way ANOVA followed by Bonferroni's post-test. Correlations were calculated by Spearman's rank test.

M-MDSCs were identified by flow cytometry as shown in Figure S1 and their suppressive capacity on CD8^+^ T cell proliferation was confirmed by a functional assay (Figure S2). The frequencies of M-MDSCs in peripheral blood of NT patients was compared with that of HC and shown to be significantly higher in HCV-infected patients (mean percentages ± SEM: 2.6 ± 0.2 and 0.8 ± 0.1 in NT and HC, respectively, *P* < 0.0001, Figure 1A). To understand if M-MDSC number perturbation was affected by the patients' virological and clinical characteristics, we next analyzed the percentages of M-MDSCs in NT subjects stratified by HCV genotype, age or sex. However, none of these factors influenced M-MDSC frequencies (Figure 1B), which were not correlated with HCV RNA viral loads, AST, and ALT values (Figure 1C). Together, these data indicate that M-MDSCs accumulate during HCV-chronic infection independently of age, sex, HCV viral load or genotype, and disease severity.

### Restoration of Quantitative and Qualitative M-MDSC Alterations Appears Late After Viral Clearance

To evaluate whether the use of IFN-free antiviral therapies was associated with immunomodulatory effects and restoration of immune functions to physiological levels, the frequency of M-MDSCs was compared among chronically HCV-infected patients untreated (NT), under therapy (T), with cleared infection (SVR) and HC. Despite HCV RNA viral loads and clinical symptoms were already restored during therapy (Table S1), the percentages of M-MDSCs were comparable among NT, T and SVR groups and significantly higher if compared to HC (Figure 1A; *P* < 0.0001, *P* < 0.0001, and *P* < 0.05, respectively), suggesting that viral clearance *per se* is not readily associated to the reduction of M-MDSC numbers. As T patients showed similar features of NT and SVR groups, they were not considered for further analysis.

To determine whether DAA treatments affected the qualitative properties of M-MDSC, we then assessed the production of reactive oxygen species (ROS), which is a suppression mechanism used by MDSCs, and their metabolic fitness by measuring the mitochondrial membrane potential (ΔΨM), considered a surrogate marker of mitochondrial activity (Zorova et al., 2018). As shown in Figure 1D, ROS levels were comparable across the study groups. Conversely, the ΔψM levels of M-MDSCs were significantly higher in NT patients compared to HC (Figure 1E; *P* < 0.05). Interestingly, SVR patients showed intermediate M-MDSC ΔΨM levels between HC and NT, which may suggest a normalization of this parameter after DAA therapies (Figure 1E).

**Figure 2:**
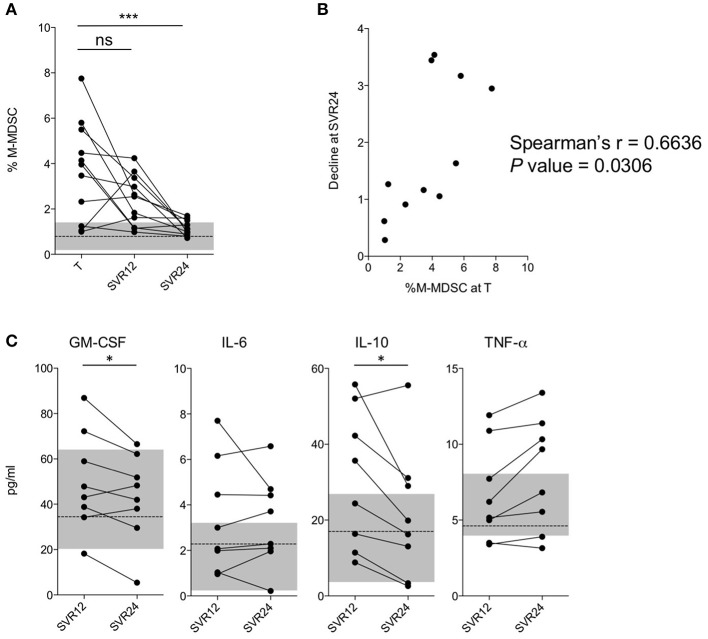
Analysis of M-MDSC frequencies in a longitudinal cohort. **(A)** Percentage of M-MDSCs in subjects with HCV-chronic infection (nested longitudinal cohort, *n* = 11) during treatment (T), at SVR12, and at SVR24. The dashed line represents the median value of M-MDSC percentages in HC (*n* = 38), and the gray area the interquartile range. **(B)** Correlation between the percentage of M-MDSCs during treatment (T) and the ratio between the percentage at time T and SVR24 (longitudinal cohort, *n* = 11). **(C)** Cytokines levels in subjects with HCV-chronic infection at SVR12 and at SVR24 (longitudinal cohort, *n* = 8). The dashed line represents the median concentrations in HC (*n* = 14), and the gray area the interquartile range. ns, not significant, ^*^P < 0.05, ^***^P < 0.0001 calculated by one-way repeated measures ANOVA followed by the Bonferroni's post-test **(A)** or by Wilcoxon signed-rank test **(C)**. Correlations were calculated by Spearman's rank test **(B)**.

To better evaluate the effects of IFN-free therapies on M-MDSC numbers, we performed a nested longitudinal study. Eleven patients (Table S3) undergoing DAA treatments (T) were followed up and blood samples collected at 12 (SVR12) and 24 (SVR24) weeks after viral clearance. Interestingly, subjects showed very low percentages of M-MDSCs at SVR24 (Figure 2A; *P* < 0.0001), with values comparable to those observed in HC. As shown in Figure 2B, the decline directly correlated with the number of M-MDSCs measured at enrollment (i.e., during therapy T). Consistently, a significant decrease of GM-CSF and IL-10, both considered involved in M-MDSC development was observed between SVR12 and SVR24 (Figure 2C). However, the same was not observed for IL-6 and TNF-α, that are also related to M-MDSC accumulation, nor for other cytokines (Figure S3).

Altogether, these data suggest that the restoration of M-MDSC numbers to physiological levels occurs fairly slowly after viral clearance, considering that it was observed 6 months from the end of the therapeutic protocol.

### DAA Therapies Revert T Cell Abnormalities Only Partially

The increase of M-MDSCs is known to be associated with the suppression of T cell functions (Tacke et al., 2012; Cai et al., 2013). As chronically HCV-infected patients, further to have an impaired HCV-specific immunity, are also characterized by reduced responses to heterologous infections (Moorman et al., 2011), extrahepatic tumors (Pol et al., 2018) and vaccines (Buxton and Kim, 2008), we determined the effects of HCV infection and DAA treatments on the whole T cell compartment. To this aim we analyzed the frequencies of different T lymphocyte subsets without considering epitope-specific immune responses.

**Figure 3:**
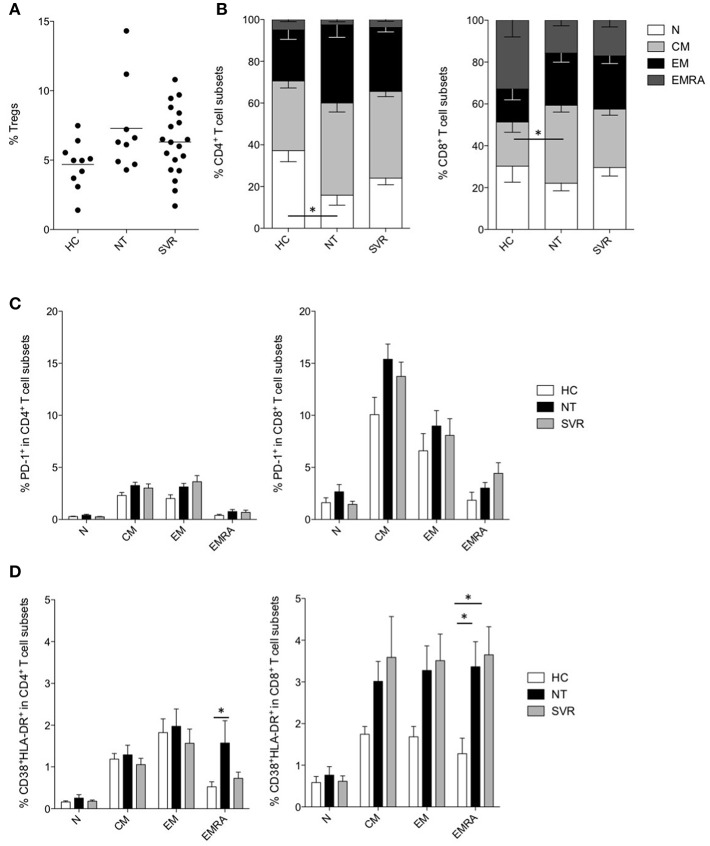
Effect of DAA-based therapies on T cells. **(A)** Percentage of Tregs in healthy controls (HC, *n* = 10) and in subjects with HCV-chronic infection, not-treated (NT, *n* = 9) and sustained virological responders (SVR, *n* = 19). **(B)** Percentage of naïve (N, CD45RA^+^CD27^+^), central memory (CM, CD45RA^−^CD27^+^), effector memory (EM, CD45RA^−^, CD27^−^) and terminally differentiated effector memory (EMRA, CD45RA^+^CD27^−^) in CD4^+^ (left panel) and CD8^+^ (right panel) T cells of HC (*n* = 10) and of subjects with HCV-chronic infection, NT (*n* = 9) and SVR (*n* = 19). **(C)** Percentage of PD-1^+^ cells among different CD4^+^ (left panel) and CD8^+^ (right panel) T cell subsets of HC (n = 10) and of subjects with HCV-chronic infection, NT (*n* = 9), and SVR (*n* = 19). **(D)** Percentage of CD38^+^HLA-DR^+^ cells among different CD4^+^ (left panel) and CD8^+^ (right panel) T cell subsets of HC (*n* = 10) and of subjects with HCV-chronic infection, NT (*n* = 9), and SVR (*n* = 19). **(A)** Lines represent the means. **(B–D)** Results were expressed as mean ± the standard error of the mean. ^*^P < 0.05 calculated by one-way ANOVA followed by Bonferroni's post-test.

As Tregs have been suggested to be among the major responsible for the dysfunctions of T cells during chronic infections, and their development is linked to that of MDSCs (Zhai et al., 2017), we measured Tregs in the NT and SVR groups in comparison with HC. However, despite the Treg numbers were slightly higher in HCV-infected NT patients and in SVR compared to HC, results did not reach statistical significance (Figure 3A, Figure S4).

Instead, the analysis of different CD4^+^ and CD8^+^ T cell subsets (Alanio et al., 2015) revealed perturbation in the T cell compartment by HCV infection (Figure S5). In particular, a significant decrease of naïve (N, CD45RA^+^CD27^+^) CD4^+^ T cells was observed in NT patients (*P* < 0.05), and their frequency only modestly increased in SVR (Figure 3B). Within the CD8^+^ T cell compartment, we could observe significant expansion of the central memory (CM, CD45RA^−^CD27^+^) subset in the NT group (*P* < 0.05), at the expenses of the N and terminal differentiated effector memory (EMRA, CD45RA^+^CD27^−^) subsets (Figure 3B). Notably, the CD8^+^ T cell subset distribution was comparable between the NT and the SVR subjects.

HCV-infection is known to induce qualitative alterations in T cells as mechanism of immune evasion (Fernandez-Ponce et al., 2017). To assess if DAA therapies restored these abnormalities, we analyzed the exhaustion and activation profiles of different T cell subsets. The expression of the inhibitory checkpoint PD-1 was analyzed in the different CD4^+^ and CD8^+^ T cell subsets, although we did not find any significant difference between NT and SVR groups as compared to HC (Figure 3C).

As regard to immune activation, we noticed increased co-expression of CD38 and HLA-DR in the EMRA CD4^+^ and CD8^+^ T cells of HCV-infected patients (*P* < 0.05) (Figure 3D). Interestingly, although not statistically significant, all other memory CD8^+^ T cell subsets showed higher activation (Figure 3D). However, while EMRA CD4^+^ T cell activation was restored to HC levels in the SVR cohort, the same was not true for the CD8^+^ T cell compartment. Indeed, the NT and SVR subjects showed a similar co-expression of CD38 and HLA-DR which, for both groups, were significantly higher (*P* < 0.05) in the EMRA CD8^+^ T cell subset when compared to HC (Figure 3D). Taken together, these data indicate that HCV infection perturbs the distribution of T cell subsets and induces their hyper-activation, and that these phenomena are restored only partially after viral clearance.

### Effects of DAA Therapies on Immunological Alterations at 48 Weeks After Viral Clearance

**Figure 4:**
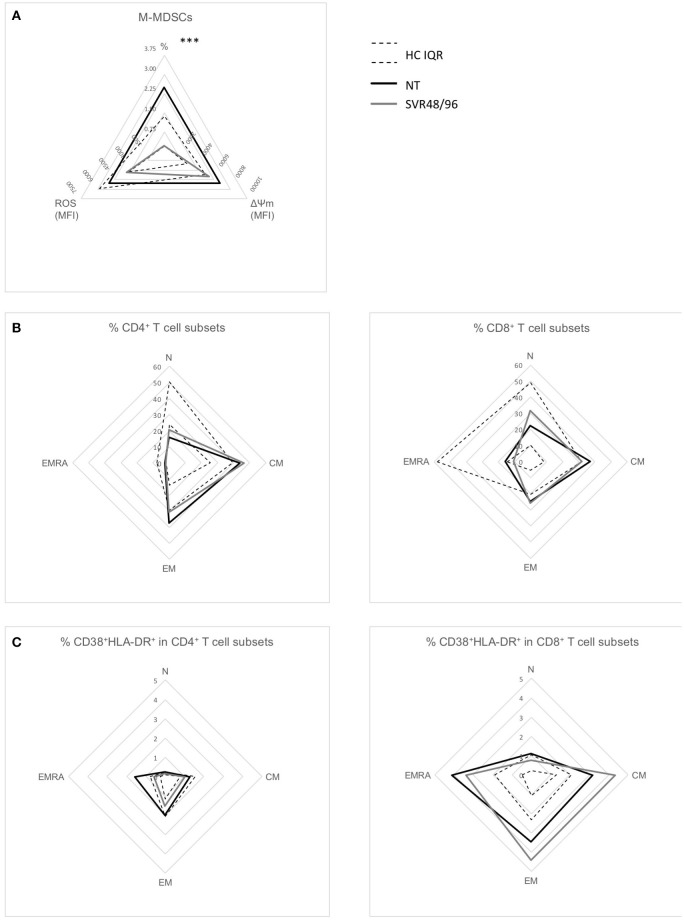
Radar plots of immunological changes at SVR48/96. **(A)** Mean percentages of M-MDSCs of subjects with HCV-chronic infection, not-treated (NT, *n* = 66), and sustained virological responders at 48 and 96 weeks (SVR48/96, *n* = 9); mean ROS content and ΔΨM levels (both expressed as MFI) in M-MDSCs of subjects with HCV-chronic infection, NT (*n* = 10), and SVR48/96 (*n* = 8). **(B)** Mean percentages of different CD4^+^ (left panel) and CD8^+^ (right panel) T cell subsets in NT (*n* = 9) and SVR48/96 (*n* = 8). **(C)** Mean percentages of CD38^+^HLA-DR^+^ on different CD4^+^ (left panel) and CD8^+^ (right panel) T cell subsets in NT (*n* = 9) and SVR48/96 (*n* = 8). Black lines represent NT subjects, gray lines SVR48/96 subjects and dashed lines represent the interquartile range (IQR) of values from healthy controls (HC). ^***^P < 0.0001 calculated by Mann-Whitney *U-*test.

As our data indicate that treatment with DAAs is associated with restoration of M-MDSC numbers at SVR24, we further analyzed our dataset focusing only on sustained virological responders enrolled at week ≥48 (SVR48/96) to assess if immunological abnormalities found in NT patients were normalized at these late time points. Consistently with the previous results, percentages of M-MDSCs were significantly lower in SVR48/96 subjects compared to NT (Figure 4A; *P* < 0.0001), whereas no significant differences were observed regarding ROS production and ΔΨM.

The SVR48/96 group showed only a minimal amelioration of T cell parameters previously observed to be altered in NT patients; indeed, percentages of N CD4^+^ T cells were slightly increased and percentages of CM CD8^+^ T cells slightly decreased (Figure 4B) at 48/96 weeks after viral clearance, but not reaching statistical significance. Similarly, EMRA CD8^+^ T cell hyperactivation was not reverted to physiological levels in SVR48/96 subjects (Figure 4C).

These data further confirm that DAA therapies slowly normalize the levels of circulating M-MDSCs, while the abnormalities in T lymphocytes persist and are partially restored after almost 2 years from the end of therapy and viral clearance.

## Discussion

In the last years, few studies suggested that HCV promotes the increase of MDSCs (Tacke et al., 2012; Cai et al., 2013; Ning et al., 2015; Pang et al., 2016). Consistently, we observed their accumulation in individuals with chronic HCV infection. The dynamics underlying this phenomenon are, however, only partially known and controversial. Indeed, it has been reported that the frequency of MDSCs correlates with the clinical biochemical parameters of HCV patients, including RNA viral load and the level of ALT and AST, which reflect liver injury (Cai et al., 2013). However, we and others (Ning et al., 2015) did not observe such correlations, suggesting that mechanisms explaining the HCV-induced increase of MDSCs is a complex phenomenon deserving further investigations, probably due to the infection *per se* rather than its clinical outcome. Indeed, HCV core protein induces MDSCs (Tacke et al., 2012) through the PI3K pathway and autocrine cytokines, such as IL-10, IFN-β, and TNF-α (Pang et al., 2016). In addition, signals that acts through STAT3 prompt MDSC differentiation and accumulation (Condamine and Gabrilovich, 2011). These signals include GM-CSF and IL-6 (Lechner et al., 2010), both induced by HCV infection (Malaguarnera et al., 1997; Chusri et al., 2016).

The accumulation of M-MDSCs was not reverted in T and SVR groups, as instead recently shown by others (Li et al., 2018), indicating that DAA treatments do not have any direct effect on their number. Sub-analysis on most frequent DAA regimens suggested that this effect is not dependent by the type of therapy (not shown). Notably, our results are in line with those reported by Tumino et al. (2017) in HCV/HIV co-infected patients. However, the results of the longitudinal cohort revealed a significant decline of the frequency of M-MDSCs starting from 24 weeks after viral clearance. The apparent discrepancy between the cross-sectional and the nested longitudinal cohorts may depend by the fact that more than half of SVR patients, in the cross-sectional study, were at SVR12. At this time point, even in the longitudinal cohort, a significant decline was not observed. However, when we just focused on SVR48/96 enrolled within the cross-sectional study, their M-MDSCs percentages were significantly lower compared to those of NT (Figure 4A). Their slow reduction might be explained by two mechanisms, such as a long half-life of M-MDSCs, surviving also after viral clearance, although to our knowledge data regarding the *in vivo* half-life of M-MDSCs in chronic infections are not available. Alternatively, it is plausible that the presence of soluble factors (Pang et al., 2016), persisting at high levels for some weeks after viral clearance, promote the survival of M-MDSCs. Consistently, we noticed a significant decline of GM-CSF and IL-10 between SVR12 and SVR24, that mirrored the decline in M-MDSCs observed between these time points. Others described a decreased frequency of M-MDSCs after 4 weeks of treatment with IFN-based therapy (Cai et al., 2013), probably because IFN itself impairs M-MDSC differentiation (Dangi et al., 2018).

MDSCs of HCV-infected individuals, although poorly characterized compared to those derived from cancer patients, are able to inhibit T cell proliferation mainly by means of ARG1 (Cai et al., 2013) and ROS production (Tacke et al., 2012). We observed high ARG1 levels in M-MDSCs, irrespectively of the study group (not shown), and comparable ROS production between HC, NT and SVR subjects, suggesting that HCV infection and the subsequent treatment do not affect the suppressor pathways used by M-MDSCs. However, M-MDSCs from NT patients showed very high levels of ΔΨM, that may derive by the increased activity of the tricarboxylic acid (TCA) cycle and electron transport chain (Zorova et al., 2018) or by high levels of fatty acid oxidation (FAO) (Schonfeld et al., 2010). Notably, FAO is crucial for M-MDSC suppressor functions (Hossain et al., 2015), that are also fueled by the TCA cycle (Hammami et al., 2012). Therefore, the enhanced levels of ΔΨM observed in M-MDSCs from NT patients could reflect an ongoing suppression activity. As this is the first work assessing metabolic alterations of M-MDSCs during HCV infection, further studies are needed to deepen this aspect.

Several studies conducted in HCV infected patients reported the increased levels of Tregs, which are responsible together with MDSCs of dampening T cell responses (Barjon et al., 2015; Zhai et al., 2017). Our results showed a trend toward higher Treg levels in NT patients compared to HC, a pattern maintained also after sustained virological response in DAA-treated individuals, in line with recent studies (Langhans et al., 2017; Tumino et al., 2017). Conversely, reports showing a decrease of M-MDSCs after IFN-based therapies also showed a reduction of Treg numbers (Su et al., 2014), confirming the strong link between these two cell populations (Zhai et al., 2017) and suggesting that IFN-free and IFN-based therapies have opposite effects on their dynamics.

It has been proposed that the increase in Treg numbers occurring during chronic HCV infection is associated with higher expression of inhibitory receptors on T cell surface (Barjon et al., 2015). High levels of PD-1 on bulk CD4^+^ and CD8^+^ T cells and on HCV-specific and liver-infiltrating CD8^+^ T cells have been described during chronic HCV infection (Urbani et al., 2006; Shen et al., 2010, 2011; Su et al., 2014). In contrast, we did not find any significant increase in PD-1 levels on the different T cell subsets. Our data suggest that the increased PD-1 expression observed by others in bulk CD4^+^ and CD8^+^ T cells may be due to lymphocyte subset redistribution, such as the loss of PD-1 negative cells (like naïve T cells), and the enrichment in subpopulations with higher PD-1 levels.

With this study, we aimed also at investigating whether chronic HCV infection, and DAA treatment, could affect the overall T cell compartment. HCV-infected individuals show reduced numbers of naïve T cells and a proportional increase of memory subpopulations in both CD4^+^ and CD8^+^ T cells, and the magnitude of these alterations predicts response to therapy (Shen et al., 2010, 2011; Hutchinson et al., 2018). In agreement, we observed reduction of N CD4^+^ T cells and increase of CM CD8^+^ T cells in NT subjects. The number of N T cells may take more than 2 years after viral clearance through IFN-based therapy to reach values similar to those of HC (Alanio et al., 2015). Consistently with these observations and with a previous report (Hartling et al., 2015), the alteration of T cell subset proportion was only slightly mitigated by DAA therapies and, as already observed during HIV infection (Nicoli et al., 2016), was associated with immune activation (Shen et al., 2010). However, our results confirm these data only partially, as we show higher CD8^+^ T cell activation in the memory, but not in the naïve, compartment while only terminally differentiated CD4^+^ T cells experienced the same phenomenon. In addition, while DAA therapies normalized activation of EMRA CD4^+^ T cells, the same was not observed in the CD8^+^ compartment, confirming previous observations on bulk T cells (Hartling et al., 2015; Najafi Fard et al., 2018). Together with data reporting that DAA treatments normalize levels of soluble inflammatory markers only partially (Hengst et al., 2016; Kostadinova et al., 2018), these pieces of evidence suggest that IFN-free therapies have only minor effects on immune activation. In addition, DAA therapies do not fully restore the αβ T cell functionality (Martin et al., 2014; Wieland et al., 2017), as previously observed for γδ T cells (Ravens et al., 2018) and in animal models (Callendret et al., 2014), nor the NK compartment (Strunz et al., 2018), supporting the concept that a partial immunological recovery is achieved in chronically HCV-infected patients after IFN-free therapy. This may pose at risk SVR patients for re-infections, low response to vaccination as well as for susceptibility to other infections and tumors. For instance, recent studies describe the presence of occult HCV infection in some patients (Attar and Van Thiel, 2015; Elmasry et al., 2017) and the recurrence of HCC (Reig et al., 2016; Guarino et al., 2018; Singh et al., 2018), despite SVR after treatment with DAAs, although other reports failed in finding this association (Guarino et al., 2018; Li et al., 2019). Nonetheless, the increased levels of pro-inflammatory chemokines and hyperactivated T cells of DAA-treated patients, as well as the higher percentage of M-MDSCs, could all contribute to the development of hepatic and extrahepatic tumors (Solito et al., 2014; Makarova-Rusher et al., 2015). In addition, an impaired T cell response to heterologous infections in HCV-infected subjects has been demonstrated (Moorman et al., 2011), not restored neither by IFN-based (Barnes et al., 2009) nor by IFN-free therapies (Martin et al., 2014).

This body of evidence suggests that, although DAA therapies are very effective in clearing HCV, cured chronically HCV-infected patients do not achieve full immune reconstitution. Further studies are needed to better clarify how this lack of complete immune-restoration impacts the capacity of HCV-cured patients to arise immunity to infections, tumors, and vaccines.

## Data Availability

All datasets generated for this study are included in the manuscript and the Supplementary Files.

## Ethics Statement

The study was approved by the Ethics Committee of the Azienda Ospedaliera of Padua (Prot. n. 3136/AO/14) and conducted according to the principles expressed in the Declaration of Helsinki.

## Author Contributions

VT, FN, EE, and AC: conceptualization. VT, FN, NM, and CF: methodology and investigation. VT and FN: formal analysis. FB, EC, and EE: patients enrollment. VT, FN, and AC: writing, review, and editing. EE and AC: supervision. GP and AC: funding acquisition.

### Conflict of Interest Statement

The authors declare that the research was conducted in the absence of any commercial or financial relationships that could be construed as a potential conflict of interest.
