# Supplementary material for: In Chronic Hepatitis C Infection, Myeloid-Derived Suppressor Cell Accumulation and T Cell Dysfunctions Revert Partially and Late After Successful Direct-Acting Antiviral Treatment
Source: Front Cell Infect Microbiol. 2019 Jun 14;9:190. doi: 10.3389/fcimb.2019.00190 (PMC6588015; doi:10.3389/fcimb.2019.00190)
Supplement: Supplementary file 1 [file Table_1.docx]

Supplementary Material

In chronic hepatitis C infection, myeloid-derived suppressor cell accumulation and T cell dysfunctions revert partially and late after successful direct-acting antiviral treatment

**Valentina Telatin, Francesco Nicoli, Chiara Frasson, Nicola Menegotto, Francesco Barbaro, Eleonora Castelli, Elke Erne, Giorgio Palù, Antonella Caputo^*^**

***Correspondence**

Antonella Caputo, cpa@unife.it

## Supplementary Figures

**Figure S1.** Gating strategy for M-MDSCs. One representative healthy control (top panels) and one representative untreated HCV-infected individual (bottom panels) are shown.

**Figure S2.** T cell suppression assay. Proliferation of CD8^+^ T cells not stimulated (NT, gray) or stimulated with anti-human CD3 and anti-human CD28 monoclonal antibodies in the absence (red) or presence (blue) of M-MDSCs was measured by CFSE staining. One representative donor is shown.

**Figure S3.** Cytokines levels in subjects with HCV-chronic infection at SVR12 and at SVR24 (longitudinal cohort, n=8). The dashed line represents the median concentrations in HC (n=14), and the gray area the interquartile range. Statistical significance was calculated by Wilcoxon signed-rank test.

**Figure S4.** Gating strategy for Tregs from CD3^+^CD4^+^ cells. CD38 and HLA-DR were used to facilitate the gating. One representative healthy control (top panels) and one representative untreated HCV-infected individual (bottom panels) are shown.

**Figure S5.** Gating strategy for CD4^+^ (top panels) and CD8^+^ (bottom panels) T lymphocytes from CD3^+^ cells. One representative healthy control (left panels) and one representative untreated HCV-infected individual (right panels) are shown. Naïve (N) T cells were defined as CD45RA^+^CD27^+^, central memory (CM) as CD45RA^-^CD27^+^, effector memory (EM) as CD45RA^-^CD27^-^ and terminally differentiated effector memory (EMRA) as CD45RA^+^CD27^-^.

## Supplementary Tables

**Table S1.** Baseline characteristics of HCV-infected and control subjects (cross-sectional study)

**Table S2.** DAA therapies of study subjects (cross-sectional study)

**Table S3.** Baseline characteristic (during therapy, T) of study subjects enrolled for the nested longitudinal study

**Table S1.** Baseline characteristics of HCV-infected and control subjects (cross-sectional study)

| **Groups** | | **HC^†^** | **HCV-infected patients** | | |
| --- | --- | --- | --- | --- | --- |
|  |  |  | **NT**^‡^ | **T^§^** | **SVR^¶^** |
| **Patients (n)** | | 47 | 75 | 53 | 40 |
| **Sex**  **(n: male/female)** | | 20/27 | 35/40 | 25/28 | 19/21 |
| **Age**  **[mean (range)]** | | 54  (32-69) | 58  (24-88) | 60  (39-83) | 54  (29-75) |
| **HCV RNA IU/mL [mean (range)]** | | NA | 2.01x10^6^  (3.8x10^3^-2.77x10^7^) | 5.10x10^1^  (0-9.90x10^2^) | 0  (0-0) |
| **AST U/L**  **[mean (range)]** | | ND | 63.62  (12-374) | 32.74  (17-75) | 27.8  (15-50) |
| **ALT U/L**  **[mean (range)]** | | ND | 78.7  (10-587) | 29.28  (10-92) | 24.5  (12-38) |
| **HCV genotype (n)** | **1** | NA | 43 | 29 | 37 |
|  | **2** | NA | 8 | 9 | 0 |
|  | **3** | NA | 15 | 9 | 1 |
|  | **4** | NA | 9 | 5 | 2 |
|  | **5** | NA | 0 | 1 | 0 |

NA, not applicable; ND, not done.

**^†^** Healthy controls

^‡^ Not treated patients

**^§^** Patients during DAA therapies

**^¶^** Sustained virological responders after DAA therapies

**Table S2.** DAA therapies of study subjects (cross-sectional study)

| **Therapy** | **T^a^ (n)** | **SVR^b^ (n)** |
| --- | --- | --- |
| Ombitasvir+Paritaprevir+Ritonavir+Dasabuvir+Ribavirin | 16 | 10 |
| Ombitasvir+Paritaprevir+Ritonavir+Ribavirin | 5 | 0 |
| Elbasvir+Granzoprevir | 0 | 3 |
| Sofosbuvir+ Ribavirin | 10 | 0 |
| Sofosbuvir+Daclatasvir+Ribavirin | 9 | 1 |
| Sofosbuvir+Ledipasvir+Ribavirin | 13 | 19 |
| Sofosbuvir+Simeprevir+Ribavirin | 0 | 7 |

^a^ Patients during therapy

^b^ Sustained virological responders after therapy

**Table S3.** Baseline characteristic (during therapy, T) of study subjects enrolled for the nested longitudinal study

| **Patients** | | | **Therapy** | **Virological and clinical parameters** | | | |
| --- | --- | --- | --- | --- | --- | --- | --- |
| **ID** | **Age** | **Sex** |  | **Viral genotype** | **HCV RNA IU/mL** | **AST U/L** | **ALT U/L** |
| 1 | 44 | F | Sofosbuvir+Ledipasvir+Ribavirin | 1b | 0 | 35 | 17 |
| 2 | 80 | F | Sofosbuvir+Ribavirin | 2 | 0 | 50 | 26 |
| 3 | 56 | F | Ombitasvir+Paritaprevir+Ritonavir  +Dasabuvir+Ribavirin | 1a | 0 | 24 | 10 |
| 4 | 60 | F | Ombitasvir+Paritaprevir+Ritonavir  +Dasabuvir+Ribavirin | 1b | 0 | 23 | 16 |
| 5 | 62 | F | Sofosbuvir+Ledipasvir+Ribavirin | 1b | 0 | 31 | 28 |
| 6 | 50 | M | Ombitasvir+Paritaprevir+Ritonavir  +Dasabuvir+Ribavirin | 1b | 0 | 29 | 18 |
| 7 | 64 | F | Ombitasvir+Paritaprevir+Ritonavir  +Ribavirin | 4 | 0 | 62 | 32 |
| 8 | 52 | F | Sofosbuvir+Daclatasvir+Ribavirin | 3a | 0 | 18 | 12 |
| 9 | 75 | F | Sofosbuvir+Ribavirin | 2A | 0 | 28 | 23 |
| 10 | 50 | M | Ombitasvir+Paritaprevir/Ritonavir  +Dasabuvir+Ribavirin | 1a | 0 | 21 | 13 |
| 11 | 47 | F | Sofosbuvir+Ledipasvir+Ribavirin | 1a | 0 | 18 | 12 |
