# Supplementary figures and images for: In Chronic Hepatitis C Infection, Myeloid-Derived Suppressor Cell Accumulation and T Cell Dysfunctions Revert Partially and Late After Successful Direct-Acting Antiviral Treatment
Source: Front Cell Infect Microbiol. 2019 Jun 14;9:190. doi: 10.3389/fcimb.2019.00190 (PMC6588015; doi:10.3389/fcimb.2019.00190)

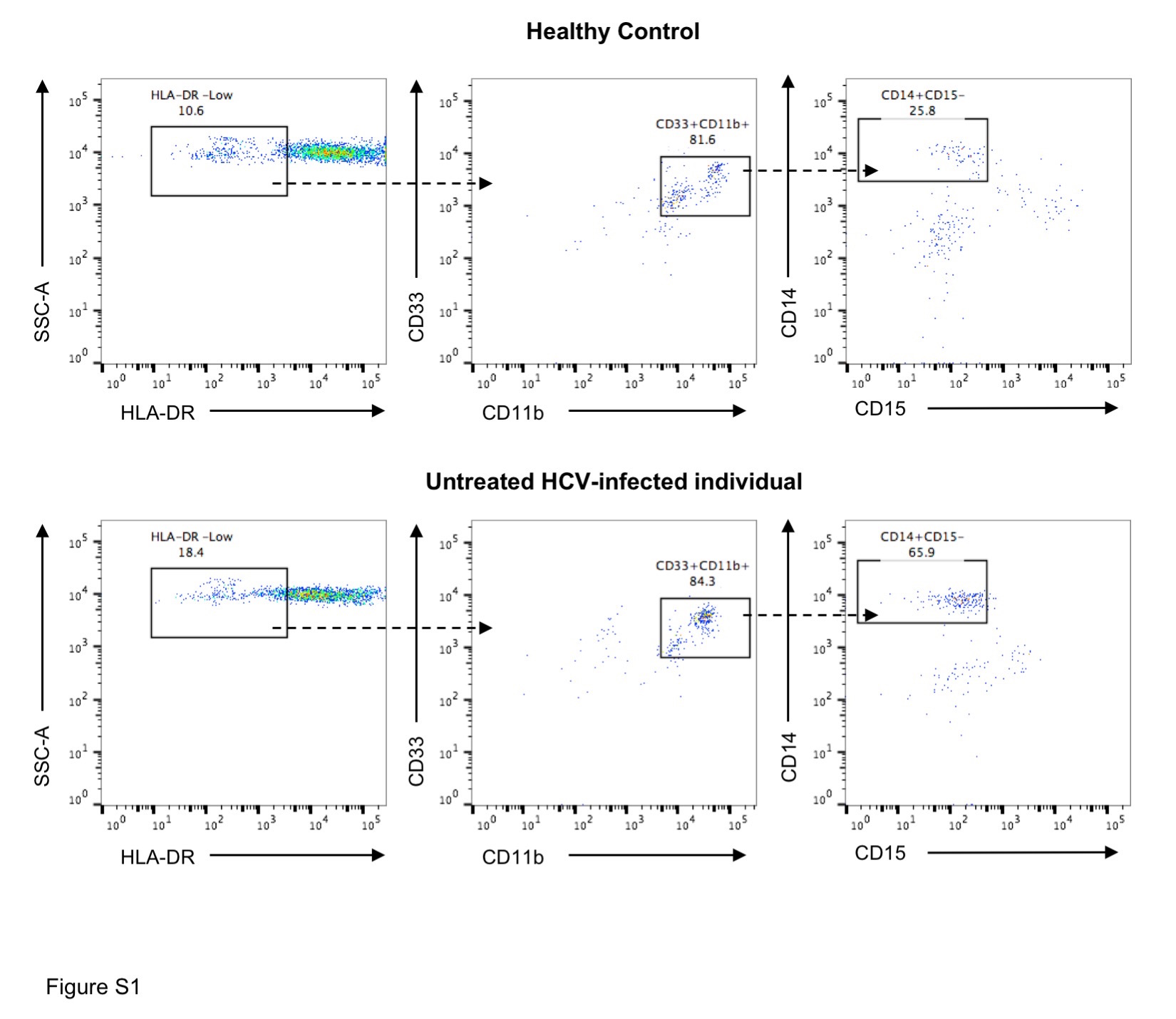

Supplement: Supplementary file 2 [file Image_1.JPEG]

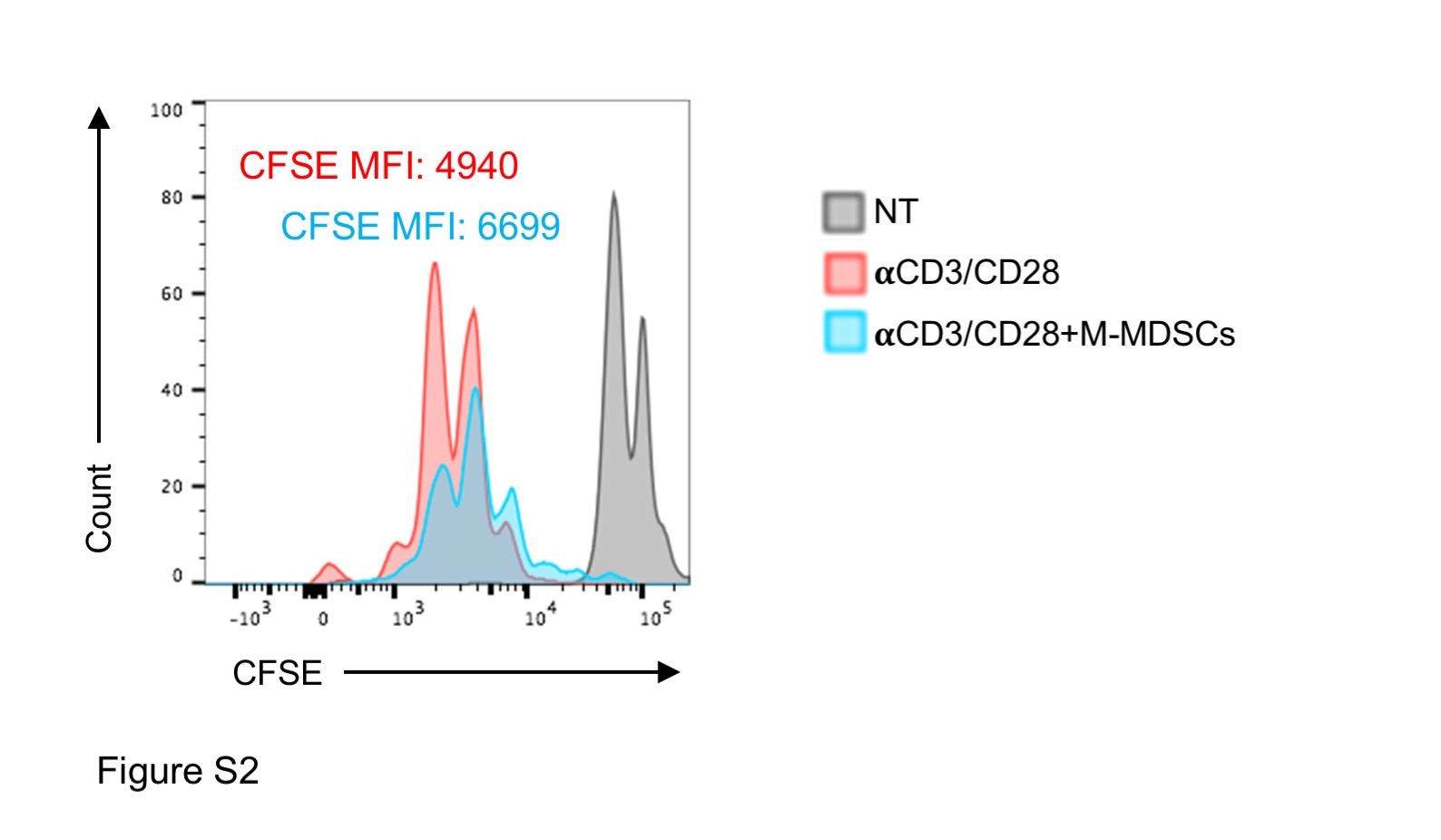

Supplement: Supplementary file 3 [file Image_2.JPEG]

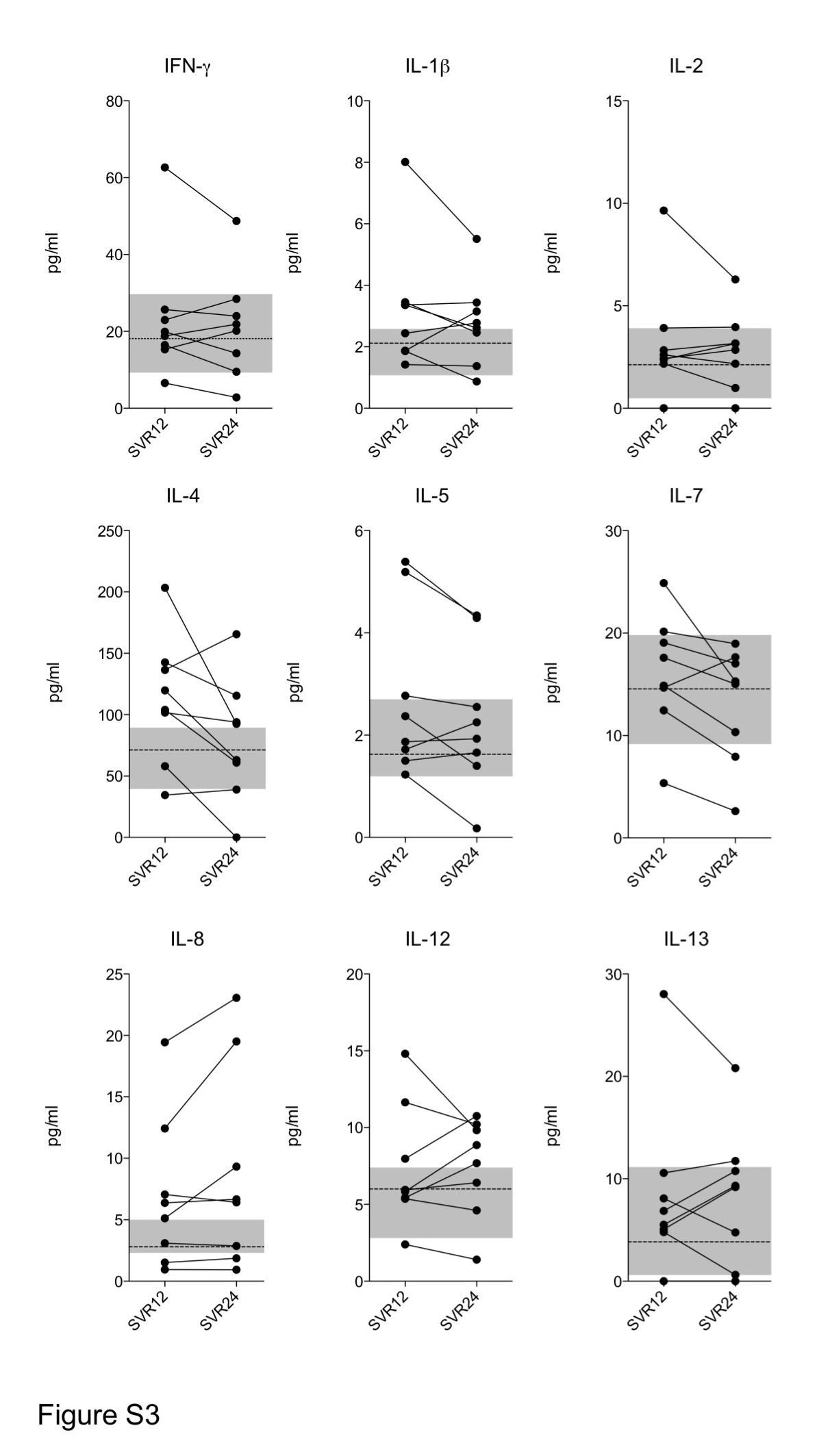

Supplement: Supplementary file 4 [file Image_3.JPEG]

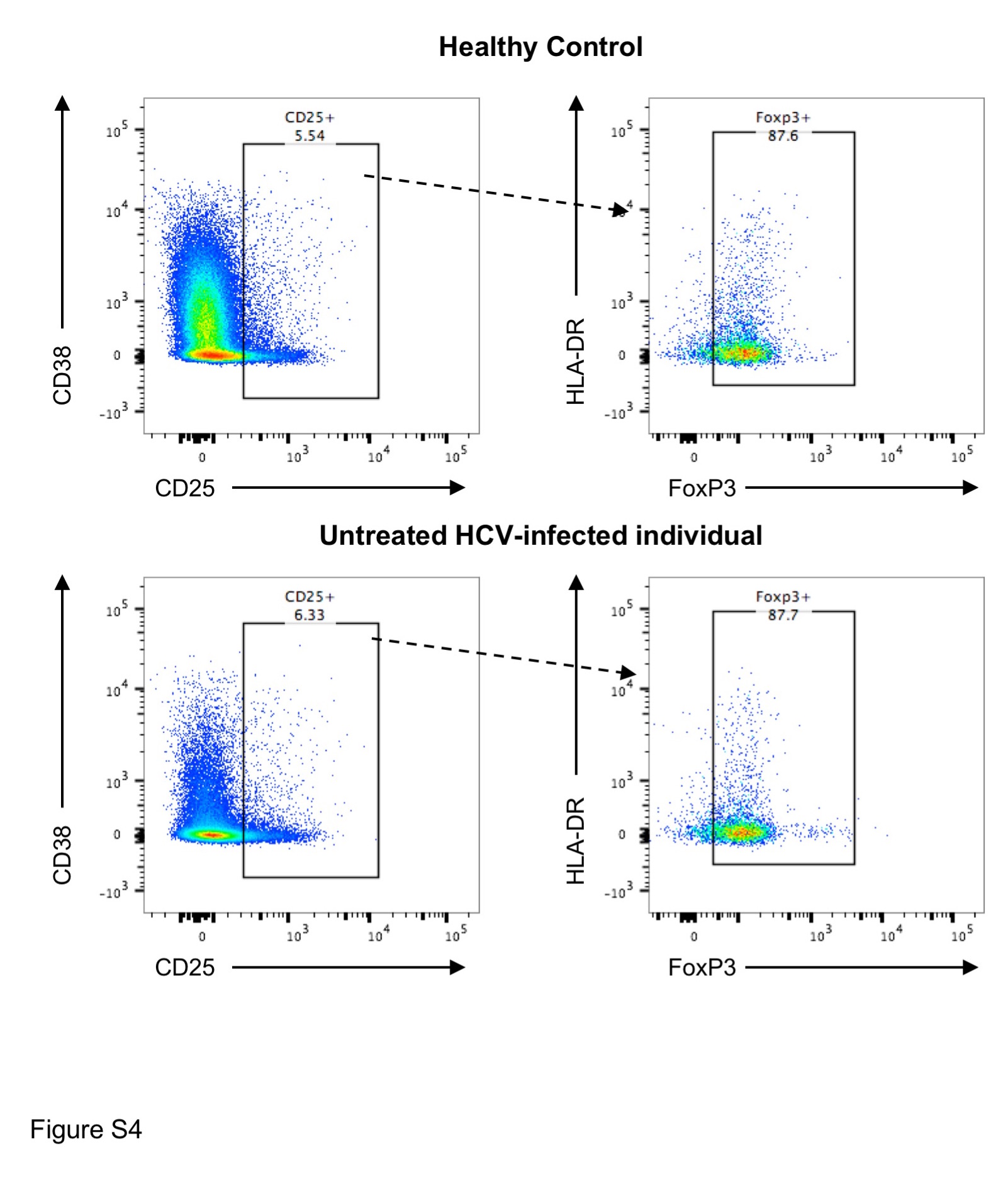

Supplement: Supplementary file 5 [file Image_4.JPEG]

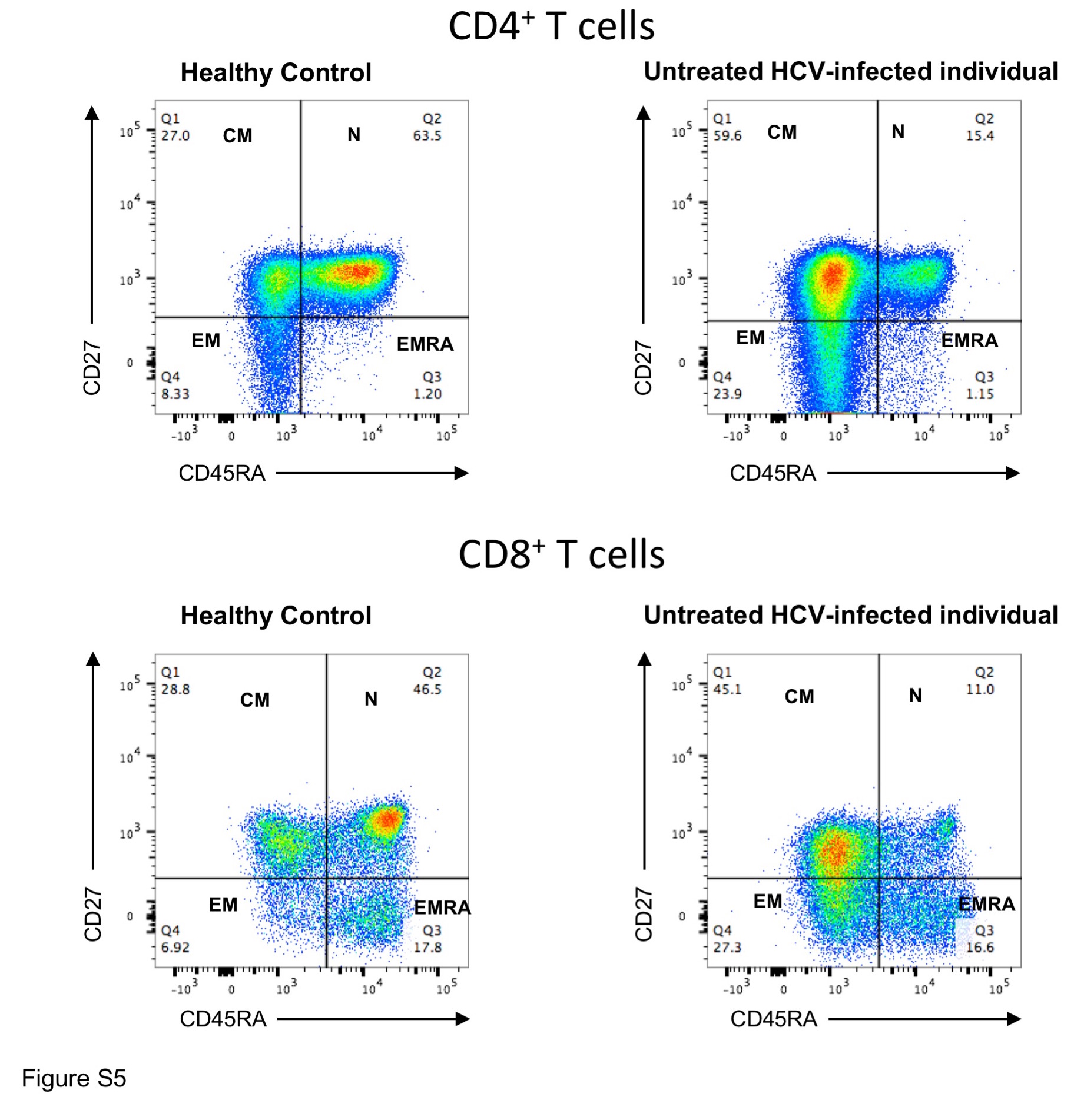

Supplement: Supplementary file 6 [file Image_5.JPEG]
